# A review of the factors influencing adoption of digital health applications for people living with dementia

**DOI:** 10.1177/20552076231162985

**Published:** 2023-03-15

**Authors:** Aoife Conway, Assumpta Ryan, Deirdre Harkin, Claire Mc Cauley, Deborah Goode

**Affiliations:** 1 School of Nursing and Paramedic Science, Faculty of Life & Health Sciences, 2596 Ulster University, Co. Derry, Northern Ireland

**Keywords:** Mobile applications, dementia, technology acceptance, technology adoption, assistive technology, apps

## Abstract

**Objective:**

Researchers have used various theories and models to understand technology adoption, however, with the growing interest and availability of mobile applications (apps) for people living with dementia, it is desirable to have a broader insight into how technology adoption may be further improved. This paper aims to explore the factors influencing the adoption of digital health applications for people living with dementia and add to the current literature on this topic.

**Methods:**

Searches were conducted in CINHAL, Web of Science, Psych Info, ProQuest Health and Medical, IEEE Xplore and Scopus. Citation searching and handsearching were used in the identification of other studies.

**Results:**

Following an assessment of relevancy, nine studies remained and are included within this review. Methodological quality was assessed using The Mixed Methods Appraisal Tool (MMAT). A thematic analysis was used for the data synthesis of included studies. Each study reported on different types of apps.

**Conclusion:**

From the synthesis of included studies, four analytic themes were identified; Theme 1: Personal and contextual factors; Theme 2: Perceived value and benefit; Theme 3: Design and content of app; and Theme 4: Digital Literacy and Confidence. People are diverse and so are their reasons for the adoption of apps. These findings provide an insight into the range of factors that impact the adoption of apps for people living with dementia. Understanding the factors that impact the adoption of mobile applications is critical to their success. These findings can be beneficial for app developers and for people living with dementia and their carers.

## Introduction

Access to mobile devices such as smartphones and tablet computers has become increasingly widespread. Many people own a smartphone and there is an increase in the ownership of multiple devices.^1^ This is not limited to the younger generation, there has also been an increase in the number of older adults using smartphones.^2^ The availability of devices has resulted in the proliferation of the time people spend engaging with digital content and the range of resources they can access. Digital health applications (apps) are among the most discussed and researched topics within healthcare innovation as they have the potential to revolutionise clinical practice.^3,4^ The number of health apps is on the rise^5^ creating new opportunities to provide multiple health functionalities in an efficient, cost-effective way by removing geographical, temporal and other barriers.^6^ The development of apps that aim to provide tools, interventions, education or communications to support health care practice is widely available for people with healthcare needs such as depression,^7^ diabetes^8^ pregnancy,^9^ cardiovascular disease,^10^ pain^11^ and needs associated with dementia.

The number of people living with dementia is increasing; this is an issue of global concern. It is suggested that around 55 million people currently have a diagnosis of dementia, and this is expected to rise to 78 million in 2030.^12^ With the increasing pressure on healthcare provision, utilising apps on smartphones or tablets may offer additional approaches to safely engage and support people living with dementia and their carers.^13,14^ There is an increased number of apps designed for people living with dementia, some of which aim to offer meaningful activities such as music,^15^ digital games,^16^ reminiscence^17^ and physical activity.^18^ Previous studies have suggested that mobile apps can be a valuable resource for people living with dementia and their carers, as they provide opportunities to deliver interventions in a person's own home.^17,19^ With the growing interest and availability of mobile apps for people living with dementia, it is desirable to have a broader insight into the acceptance of digital health apps for this population.

Understanding the factors that impact the adoption and acceptance of mobile applications is critical to their success.^20^ There is a rich body of work on technology acceptance. Researchers have used various theories and models to understand factors influencing technology acceptance, for example, Technology Acceptance Model^21^ and the Unified Theory of Acceptance and Use of Technology 2 (UTAUT2).^22,23^ Despite the availability of such models, it has been argued that the understanding of user acceptance is limited due to a variety of interpretations of what constitutes ‘technology acceptance’.^24^ Nadal et al.^24,25^ propose the Technology Acceptance Lifecycle (TAL) as a method to provide clarity on the concept. The TAL is a continuum that can anchor the definitions of technology acceptance. This continuum begins with pre-use acceptability, this stage encompasses the period before any interaction with technology occurs. The first interaction with a technology marks the end of pre-use stage and the beginning of the initial use acceptance stage. The final stage, sustained use acceptance, is when the user reaches the point of adopting the technology.

Previous research has studied the adoption of apps for other health care needs; however, little is known about the factors relevant to the adoption among people living with dementia. Therefore, this paper aims to review, critique, synthesise and update what is already known about the topic as well as identify any gaps in the literature.^26^ The following sections will explain the comprehensive methodology of this review as well as the findings. An in-depth discussion is provided on the significance of these findings in relation to what was already known and an explanation of any new insights that emerged as a result of the review. Overall conclusions will be presented as well as the study’s strengths and its implications and proposes the impact on further research and policy.

## Methods

The integrative review methodology proposed by Whittemore and Knafl^27^ was implemented to guide the process and to ensure a systematic and rigorous approach to answer the research question ‘What are the factors relevant to the adoption of apps among people living with dementia?’. This method was chosen as it allows for the inclusion of diverse methodologies and, therefore, has the potential to provide a greater understanding of what is already known about the topic.^27^

Stage 1 began with a preliminary search of relevant literature. This identified a dearth of research on the factors influencing the adoption of applications (apps) among people living with dementia. The SPIDER strategy (Sample, Phenomenon of Interest, Design, Evaluation, Research type) was used as a guide to develop a clear, focused and relevant review question and to structure the inclusion/exclusion criteria.^28^ This strategy was employed as it has been found to be suited to qualitative and mixed methods research.^29^

Stage 2 involved completing a comprehensive and rigorous search, the reviewer worked with two subject librarians within the Schools of Nursing and Computing. These librarians peer-reviewed the search strategy, search terms and choice of electronic databases.^30–32^ This helped to identify databases that were not routinely used by the authors. This also ensured the review was comprehensive and as free from bias as possible.^33^ Multiple databases covering a range of disciplines and content were searched contributing to a comprehensive and rigorous review.^34^

Search terms were identified by brainstorming the review question and completing preliminary exploratory searches.^30^ The first concept identified was dementia, and the second was the use of applications. Search terms were combined with Subject terms, MeSH headings or keyword searches depending on the database. Limiters of English language publications and a time frame of five years were applied to ensure the inclusion of relatively current and clinically relevant research and in keeping with current technology differences or trends (Table 1).^35^

**Table 1.** Search terms.

Database	Search terms	Limiters	Results
CINHAL	(MH ‘Dementia+’) (MH ‘Mobile Applications’) (Dementia or Alzheimer*) "digital health app*" or "mobile health app*" or "mhealth app*" or mhealth or ehealth or telehealth	English Language Publication date 5 years	325
Web of Science	(Dementia or Alzheimer*) AND ("digital health app*" or "mobile health app*" or "mhealth app*" or mhealth or ehealth or telehealth)	English Language Publication date 5 years	308
PsycInfo (Ovid)	Exp Dementia (Dementia or Alzheimer*) Exp Mobile applications ("digital health app*" or "mobile health app*" or "mhealth app*" or mhealth or ehealth or telehealth)	English Language Publication date 5 years	87
ProQuest Health and Medical	( Dementia or Alzheimer*) AND ("digital health app*" OR "mobile health app*" OR "mhealth apps" OR mhealth OR ehealth OR telehealth)	English Language Publication date 5 years	232
IEEE Xplore	All Metadata: "dementia OR alzheimer*" All Metadata: "digital health app*" OR "mobile health app*" OR "mhealth app*" OR mhealth OR ehealth OR telehealth	Publication date 5 years	14
Scopus	(dementia OR alzheimer* ) AND "digital health app*" OR "mobile health app*" OR "mhealth app*" OR mhealth OR ehealth OR telehealth	English Language	428

**Figure 1.**
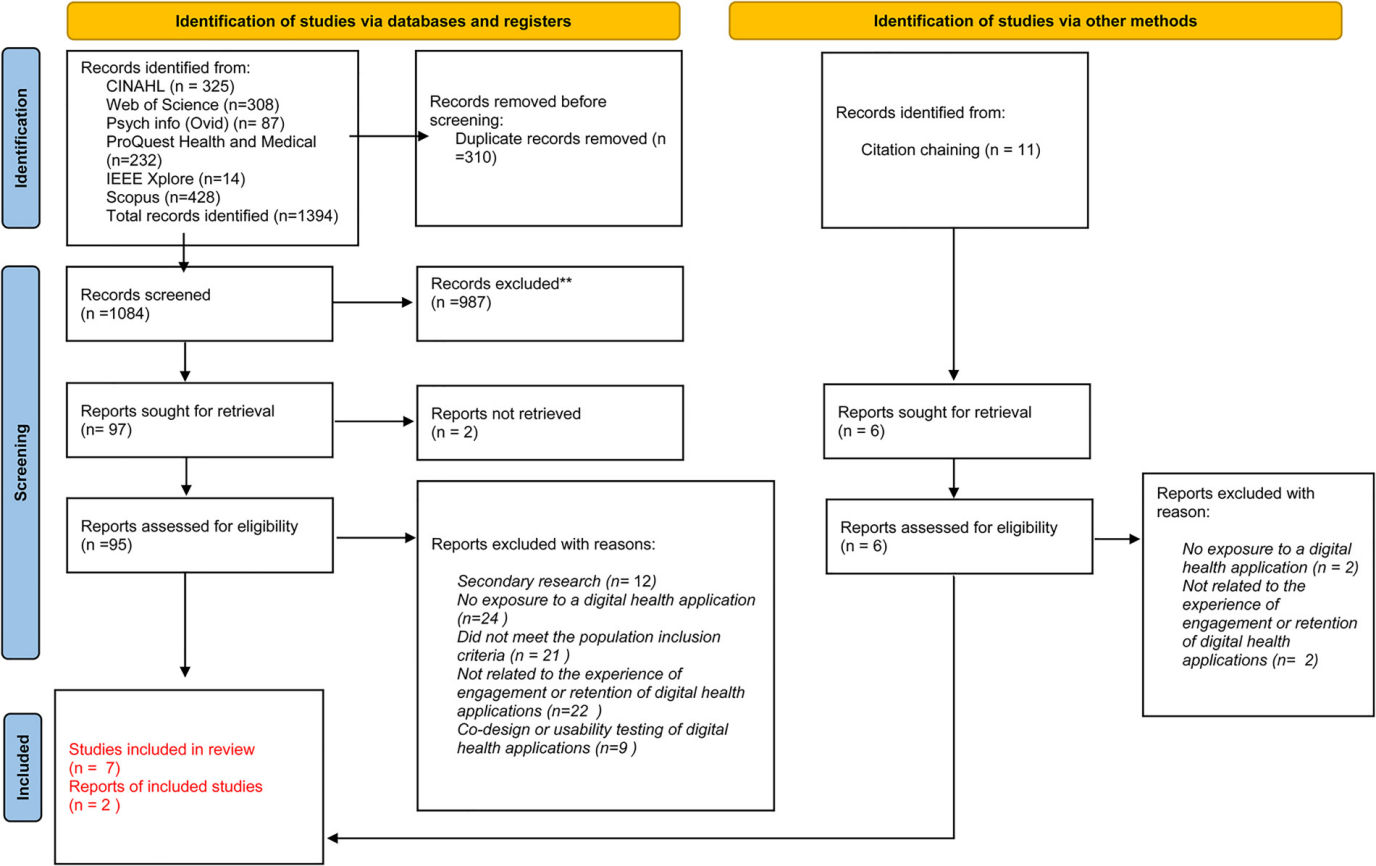
PRISMA 2020 flow diagram for new systematic reviews which included searches of databases, registers and other sources.

Exhaustive measures were used in the identification of other studies including citation searching and handsearching relevant journals and reference lists to find publications on the same or similar subject and to ensure a comprehensive review.^27^ The database Scopus aided this search. In keeping with the nature of integrative reviews the search was broad in nature^33^ and then focused on assessing relevancy using the inclusion/exclusion criteria (Table 2). Using the inclusion criteria, studies were first assessed by title and abstract. Following this, 95 studies from electronic databases and 11 studies identified via other methods were read in full-text multiple times and assessed for relevancy. The reasons for exclusion are documented in the PRISMA P Flow Diagram^36^ (Figure 1). Nine studies remained and are included within this review.

**Table 2.** Inclusion and exclusion criteria.

	**Include**	**Exclude**
**Sample**	People living with a confirmed diagnosis of dementia People living with a confirmed diagnosis of dementia and Carers (formal or informal) of people living with dementia	People living with cognitive impairments or memory loss Studies where participants cannot be separated from cognitive impairment and dementia. Studies pertaining to apps for Carers of people living with dementia
**Phenomenon of Interest**	Exposure to a digital health application	Studies pertaining to wearable technologies, sensors or gamified applications
**Study design**	Any	Studies will not be excluded due to design
**Evaluation**	Barriers or facilitators experienced by people living with dementia and carers relevant to engagement	The impact of digital health applications on outcome measurements
**Research type**	Primary research including quantitative and qualitative studies	Systematic reviews, opinion articles, editorials and abstract only available

Stage 3, as outlined by Whittemore and Knafl,^27^ was the data evaluation stage. The aim was to enhance the rigor of the review by evaluating the quality of studies chosen. The Mixed Methods Appraisal Tool (MMAT)^37^ was used as it enabled comparisons of quality to be made across various methodologies.^38^ To ensure accuracy and minimise bias, two authors (**and**) independently reviewed all the literature for quality and relevance. This review process was followed by a lengthy discussion to reach a consensus. Hong et al.^37^ discourage calculating an overall score from the MMAT ratings and recommend that the tool is used to inform the overall quality of the included studies.^37^ Both reviewers agreed that the final number of studies (*n* = 9) selected for the review were of high quality.

Stage 4 involved coding and categorising the information from the selected studies to provide an innovative synthesis of the evidence.^39^ A Data Extraction Matrix was used to extract key information from the nine studies included in the review (Table 3). A thematic analysis was used for the data synthesis of included studies. The aim of this was to identify, analyse and report patterns within data. The reviewer went through the findings of each study and selected important data and coded each line of text according to its meaning and content. Each of the codes was reviewed and similarities between the codes identified to start grouping them into descriptive categories.

**Table 3.** Data extraction.

Author/Country of study	Research aims, design and methodology	Sample characteristics	Digital health app	Key findings	Limitations	Corresponding theme
Bielsten, T., Keady, J., Kullberg, A., Lasrado, R., & Hellström, I. (2020)^41^ **Country of study:** Sweden	**Aim**: The aim of this study was to explore the experiences of using the application among couples where one partner has dementia living at home **Design:** Qualitative research design. **Setting:** Own homes **Data collection**: Semi-structured interviews (Audio recorded and transcribed) **Data Analysis:** Thematic analysis	**Participants:** 12 **Gender:** 1 female and 5 males living with dementia. 1 male 5 five female partners **Age:** People living with dementia ranged from 66–81. Age range of partners 64–83. **Diagnosis:** four people living with Alzheimer's disease, one person living with Frontotemporal dementia and one person living with dementia of unspecified origin	DemPower’, Self-management application.	**Key Findings**: couples where one partner has dementia value interventions that focus on a salutogenic approach. The application contributed to feelings of empowerment, satisfaction of couples’ achievements and a sense of support through both peers and the intervention. The focus on strengths, resources and quality of life can give couples insight into the growth of their relationship and the evolving transitions during the trajectory of dementia.	**Limitations:** authors interviewed six couples that tested the app, as there is no consensus in how many participants are required for a qualitative study, this could contribute to a narrower picture of the experience. Additionally, dyadic interviewing can result in fragmented data.	Theme 1: Personal and contextual factors Theme 2: Perceived value Theme 3: Design and content of app Theme 4: Digital Literacy and Confidence
Critten, V. and Kucirkova, N. (2019)^43^ **Country of study**: UK	**Aim**: 1) How can an iPad app facilitate the creation of multimedia personalised stories by people living with mild to moderate dementia? 2) What is the role of multimedia and personalisation in the stimulation, preservation and sharing of special memories in people living with mild to moderate dementia? **Design:** Case Study **Setting:** Club for people with dementia run by a housing association **Data collection:** one-to-one interviews over several occasions field notes and observations **Data Analysis:** Basic content analysis	**Participants:** 3 **Gender:** one female, two males **Age:** 72–94 **Diagnosis:** mild to moderate dementia (no specific diagnostic criteria reported)	Our Story’ Reminiscence app	**Key Findings** The multimedia features available with the Our Story app offer a unique opportunity for people living with dementia to store, access and generate memories, capture them in writing and audio; and the ability to continue adding to original stories. People living with dementia and other club members might not find this an easy or enjoyable process because of possible sad memories or physical constraints in using the iPad	**Limitations:** Did not collect interview data from the carers and family members. Small sample size, poor digital literacy of participants (training was provided). Findings are not generalisable to the wider population. The researcher–participant have positively influenced the outcomes in this study.	Theme 1: Personal and contextual factors Theme 2: Perceived value Theme 3: Design and content of app Theme 4: Digital Literacy and Confidence
Ekstrom, A., Ferm, U. and Samuelsson, C. (2017)^45^ **Country of study**: Sweden	**Aim**: To explore the characteristics of communication when a digital device is used in interaction with people with dementia. **Design:** Mixed methods case study. **Setting**: Dyads home **Data collection**: Video recordings. Two sets of data collected: one where the couple is interacting without communication support and one where they are using a tablet computer and the communication application. Quant: the number & length of recording sessions Qual: Communicative initiatives **Data Analysis:** the communicative initiatives made by the person with dementia have been identified and counted and compared between the two data sets.	**Participants:** One person living with dementia and husband **Gender:** Female (person living with dementia) and male (husband) **Age:** 52 (person living with dementia) Age of husband is unknown **Diagnosis:** Alzheimer's disease	GoTalk NOW. Developed for people with communicative problems and can be individually designed with personal pictures, video clips and digitised and synthetic speech	**Key Findings:** Results indicate that the amount of interactive actions and the number of communicative actions seem to increase with the use of the communication application.	**Limitations:** Small sample size and use of a single app. Activities were being recorded to some extent influenced the couple's communication. This might have affected both the amount of communicative activities as well as the character of these activities	Theme 1: Personal and contextual factors Theme 2: Perceived value Theme 4: Digital Literacy and Confidence
Gall, D., Pressler, J., Hurtienne, J. and Latoschik, M.E. (2020)^46^ **Country of study:** Germany	**Aim**: no clear aim. Researchers explore how self-organising knowledge management affects the quality of person-centred care. **Design**: Field study **Setting**: two care homes for people living with dementia **Data collection:** Semi-structured in-depth telephone interviews after the six-month intervention period. Subsequently, semi-structured focus groups were conducted in each facility. **Data Analysis:** Thematic analysis	**Participants:** 8 residents, 8 significant others and 6 caregivers **Gender:** Unknown **Age:** Unknown **Diagnosis**: ‘Severe dementia’ – Specific diagnosis of people living with dementia unknown	CareShare, a collaborative communication system.	**Key Findings** participants reported that the system increased the quality of person-centered care, reduced communication gaps, increased the task satisfaction of caregivers and the well-being of significant others	**Limitations:** opinions of the self-selected sample do not necessarily generalise to other significant others or caregivers. Feedback could be subject to positivity bias: participants may have favoured positive over negative memories.	Theme 1: Personal and contextual factors Theme 2: Perceived value Theme 3: Design and content of app Theme 4: Digital Literacy and Confidence
McAllister, M., Dayton, J., Oprescu, F., Katsikitis, M., & Jones, C. M. (2020).^44^ **Country of study:** Australia	**Aim**: explore (1) barriers and facilitators to Memory Keeper's use with persons with dementia in a long-term care facility, (2) the benefits (or otherwise) of the application when used in this setting and (3) the potential use and incorporation of Memory Keeper within the care currently provided in this setting **Design:** Pilot study **Setting**: dementia wing of a long-term care facility **Data collection:** field notes (recording peoples’ reactions and interactions to the intervention), focus groups and one individual interview were conducted during the trial **Data Analysis:** Framework Analysis	**Participants:** 3 people living with dementia. 6 significant others. One died **Gender:** 2 Male 1 Female **Age:** 76–87 **Diagnosis:** Lewy body Dementia, Alzheimer's, Dementia (non-specified)	Memory Keeper: A digital application to improve engagement with people with dementia in long-term care	**Key Findings** The study reported family members felt the Memory Keeper app was valuable as it helped improve the quality of engagement they had with a person with dementia and made visits more enjoyable. Recommendations were to engage people with dementia as early as possible to populate the app with personalised content and provided more training to people with dementia, their families and staff in the long-term care facility	**Limitations:** Small sample limits the generalisability of the findings Participants could have exaggerated their positive feedback and experiences and minimised any negative experiences in response to their increased familiarity with staff member collecting data	**_**
Øksnebjerg, L., Woods, B., Ruth, K., Lauridsen, A., Kristiansen, S., Hoist, H.D. and Waldemar, G. (2020)^42^ **Country of study:** Denmark	**Aim**: This study aimed to (1) evaluate the applicability and usability of an app, tailor-made for people with dementia; (2) explore factors affecting adoption; (3) explore the possible influence of caregiver involvement; and (4) contribute to process evaluation of the intervention. **Design**: Mixed methods design **Data collection:** Qualitative: A Web-based survey (including the USEdem questionnaire) **Quantitative data**: Baseline characteristics and log data **Data Analysis:** Quantitative data were analysed using IBM SPSS Statistics version 22. Non-parametric chi-square tests Kruskal–Wallis tests, Mann–Whitney *U* tests or Fisher exact tests were used as appropriate. Tests of significance were performed 2-tailed. Qualitative data constant comparison analysis	**Participants:** 112 participants and 98 caregivers **Gender:** Female:43 Male:69 Caregiver Female: 59 Male: 28 **Age:** 39–86 **Diagnosis:** Alzheimer disease – 65 Vascular dementia – 2 Dementia with Lewy bodies – 1 Frontotemporal dementia – 3 Mild cognitive impairment – 0 *Other – 27 *Unresolved – 5 *(Other = For example, stroke, Huntington disease and unspecified dementia diagnosis. Unresolved – Participants who were not diagnosed at the time of inclusion.)	ReACT app: a calendar that interacts with other features (eg, diary notes, contacts, checklists and memos).	**Key Findings:** The study provided insight into the importance of timely introduction and caregiver support for adoption of AT among people with dementia. It also underlined the high complexity of personal and contextual factors that influence adoption.	**Limitations:** Only people who have sought an examination and had contact with a memory clinic were included, staff could have been biasing inclusion A number of participants did not have a dementia diagnosis; hence, describing participants as a group of people with dementia could be considered imprecise. The app could only be used on a series of tablets (iPads) This excluded potential participants who had access to other kinds of tablets. Applying a survey to this group of end users causes limitations	Theme 1: Personal and contextual factors Theme 2: Perceived value Theme 3: Design and content of app Theme 4: Digital Literacy and Confidence
Rai, H.K., Prasetya, V.G.H., Sani, T.P., Theresia, I., Tumbelaka, P., Turana, Y., Schneider, J. and Orrell, M. (2021)^47^ **County of study:** Indonesia (urban and rural area)	**Aim**: explore the attitudes of people with dementia, carers and healthcare professionals towards the iCST application and the use and implementation of related technology in their daily lives **Design:** Mixed-method study **Data collection**: Semi-structured focus group discussions, usability and acceptability questionnaire, a short testing session of the iCST application and an expert meeting with healthcare professionals. **Data Analysis:** Thematic analysis	**Participants:** People living with dementia – 6 Carers – 12 healthcare professionals – 21* Gender: People living with dementia – 60–83 Carers – 35–72 healthcare professionals – unknown **Age:** People living with dementia – 60–83 Carers – 35–72 healthcare professionals – unknown Diagnosis: *( 7 nurses, 6 social workers, 3 medical doctors, 2 care home staff members, 1 psychologist, 1 community leader and 1 researcher. Their experience with working in dementia care ranged from two months to 33 years.)	iCST-Individual cognitive stimulation therapy application	**Key Findings:** Attitudes towards technology were positive but lack of experience, difficulties with operating devices and a limited infrastructure to support technology were described as barriers. The iCST application was seen as an interesting tool to support mental stimulation. Compared with people with dementia, carers were more willing to use the application and rated its usability higher. Healthcare professionals were positive about the interactive features of the application and judged that it could be useful within the family context.	**Limitations:** Small sample. More carers than people with dementia participated in this study. At times in this study, people with dementia did not fully participate in the discussion and required lots of support from their carers. These factors may have limited the richness of the data. There were limited activities that participants could try-out due to differing cultural contexts and language barriers. Therefore, participants were not able to comment on the full application.	Theme 1: Personal and contextual factors Theme 2: Perceived value Theme 3: Design and content of app Theme 4: Digital Literacy and Confidence
Ryan, A.A., McCauley, C.O., Laird, E.A., Gibson, A., Mulvenna, M.D., Bond, R., Bunting, B., Curran, K. and Ferry, F. (2020)^17^ **Country of study**: UK	**Aim**: The aim of this qualitative study was to explore the impact of a home-based, personalised reminiscence programme facilitated through an iPad app (InspireD) on people living with dementia and their family carers. **Design:** Qualitative study **Data collection:** Semi-structured interviews (recorded) **Data Analysis:** Thematic analysis	**Participants:** People living with dementia – 15 Family carers – 17 **Gender:** People living with dementia Female:6 Male: 9 Family carers Female: 13 Male: 4 **Age:** People living with dementia: 61–94. Carers: 31–85. **Diagnosis:** mild to moderate dementia	InspireD reminiscence app.	**Key Findings:** six key themes emerged related to usability; revisiting the past; home use; impact on the person living with dementia; gains and abilities and impact on relationships. These themes highlighted the impact of the reminiscence experience at an individual and relationship level for people living with dementia and their carers. The findings of this study indicate that a more individualised approach to reminiscence, facilitated using a tablet device and preceded by a period of reminiscence and IT training, has the potential to generate a positive impact on people living with dementia without negative consequences for family caregivers	**Limitations:** The research team formed a relationship with participants and this may have influenced the findings. A marginally higher number of carers participated in the interviews	Theme 1: Personal and contextual factors Theme 2: Perceived value Theme 3: Design and content of app Theme 4: Digital Literacy and Confidence
Tyack, C., Camic, P. M., Heron, M. J., & Hulbert, S. . (2017)^40^ **Country of study:** UK (participants from rural and urban areas)	**Aim**: 1. How does viewing art on a tablet-style computer impact the well-being of people with dementia? 2. What are informal caregivers’ impressions of this activity's impact on the people with dementia they care for? 3. How does a person with dementia experience viewing art on a tablet style computer? **Design**: Mixed-methods exploratory study. **Setting**: Not clear **Data collection:** QoL-AD scale, three visual analogue (VAS) subscales measuring happiness, wellness and interestedness and interviews. **Data Analysis:** statistical and Thematic analysis.	**Participants:**12 people with dementia and their 12 informal caregivers **Gender:** Person living with dementia: Men = 8, female = 4 **Age:** Person living with dementia: 64–90 Caregivers: 48–77 **Diagnosis:** formal diagnosis of dementia (criteria not disclosed	App divided into objects, paintings and photography (>100 images from three London museums and collections from a photographer and a painter	**Key Findings:** The study findings revealed that the well-being subdomains generally increased with number of sessions a person with dementia had with the art-based app. qualitative findings included changes in cognition, behaviour, mood and relationships. These changes tended to be viewed positively	**Limitations:** small sample size The lack of a control group makes it difficult to determine whether the impacts on well-being were directly related to the app or other factors Several different statistical analyses were run increasing the possibility of Type-I errors. is also possible that the technology of using a tablet computer was an equivalent intervention to the art itself, although this was not evident in the thematic analysis of interview data.	Theme 1: Personal and contextual factors Theme 2: Perceived value Theme 3: Design and content of app Theme 4: Digital Literacy and Confidence

## Findings

### Characteristics of included studies

Nine studies were included in the review. All studies took place from 2017 to 2021 across six countries. Studies included in this review are mainly Global North countries (*n* = 8).^17,40–46^ Beyond this, few studies describe the geographic area (rural/urban)^40,47^ or the socio-economic setting (low and middle-income countries, high-income countries). Study participants varied in terms of gender, age, living arrangements (nursing or residential home/own home/cohabiting/hospital) and formal and informal carers (Table 3). It is recognised that the settings of these studies may affect the delivery of the intervention and its outcomes. Each study reported on different types of applications (apps) including apps for viewing art^40^ self-management,^41,42^ reminiscence,^17,43,44^ communication^45,46^ and cognitive stimulation therapy.^47^ Most of the apps were used on tablet devices. From the synthesis of included studies, four key themes were identified (Table 4):

**Table 4.** Themes and findings.

Theme	Findings	Pertaining studies
*Theme 1: Personal and contextual factors*	Health status of the person living with dementia impacted their usage of the app.	Tyack et al.^40^ Øksnebjerg et al.^42^ McAllister et al.^44^
	The importance of introducing an app at the correct time.	Øksnebjerg et al.^42^ Critten and Kucirkova^43^
	Support from carers.	Ryan et al.^17^ Tyack et al.^40^ Øksnebjerg et al.^42^ Critten and Kucirkova^43^ McAllister et al.^44^ Gall et al.^46^ Rai et al.^47^
	Challenge for carers.	Tyack et al.^40^ Ekstorm et al.^45^ Gall et al.^46^
	How an app integrates into everyday life for the person living with dementia.	Øksnebjerg et al.^42^ McAllister et al.^44^ Rai et al.^47^
	Considering diverse user backgrounds when introducing an app.	Rai et al.^47^
*Theme 2*: *Perceived value*	App adds value to their day-to-day life, and it is perceived as useful.	Ryan et al.^17^ Tyack et al.^40^ Bielsten et al.^41^ Critten and Kucirkova^43^ McAllister et al.^44^ Rai et al.^47^
	Meaningful engagement.	Ryan et al.^17^ Bielsten et al.^41^ Critten and Kucirkova^43^ McAllister et al.^44^ Gall et al.^46^ Rai et al.^47^
	Increased communication.	Ryan et al.^17^ Tyack et al.^40^ Critten and Kucirkova^43^ McAllister et al.^44^ Ekstorm et al.^45^ Gall et al.^46^ Rai et al.^47^
*Theme 3: Design and content of app*	App content.	Bielsten et al.^41^ Øksnebjerg et al.^42^ Rai et al.^47^
	Strengths-based app.	Ryan et al.^17^ Bielsten et al.^41^
	Personalised app.	Ryan et al.^17^ Tyack et al.^40^ Øksnebjerg et al.^42^ Critten and Kucirkova^43^ McAllister et al.^44^ Gall et al.^46^ Rai et al.^47^
	App ease-of-use.	Ryan et al.^17^ Critten and Kucirkova^43^ McAllister et al.^44^
	Need for clear instructions.	McAllister et al.^44^ Gall et al.^46^ Rai et al.^47^
*Theme 4: Digital literacy and confidence*	The ability to operate digital technology.	Ryan et al.^17^ Øksnebjerg et al.^42^ McAllister et al.^44^ Gall et al.^46^ Rai et al.^47^
	A fear of failure or a lack of confidence.	Ryan et al.^17^ Critten and Kucirkova^43^ Gall et al.^46^ Tyack et al.^40^ Ekstorm et al.^45^
	Carers’ digital literacy.	Ryan et al.^17^ McAllister et al.^44^
	Support with digital literacy.	Rai et al.^47^

### Theme 1: Personal and contextual factors

Each of the studies highlighted a range of personal and contextual features relevant to the adoption of apps. A notable finding was how the health status of the person living with dementia impacted their usage of the app. Several studies^40,42,44^ described how engagement with an app was dependent on the condition of the person living with dementia and the stage of their dementia journey. Depending on their health condition, challenges such as physical dexterity,^40,43^ visual impairments,^40,47^ deterioration in cognition^43^ and mood variability^40^ impacted user engagement. The importance of introducing an app at the correct time is clear as studies highlight non-usage when introduced too early or too late, at a stage where the participant was ‘no longer able to learn how to use it or had lost the ability to use a touchscreen device’^42 (p.12),43^.

Many studies highlighted the value of carers support when using an app that is designed for a person living with dementia.^17,40,42,43,46,47^ Rai et al.^47^ found support from carers helped to overcome some of the barriers preventing people living with dementia using apps. Øksnebjerg et al.^42^ similarly found support from carers had a significant influence on whether participants adopted the ReACT app; however, they also noted that some participants adopted an app with minimal or no involvement from carers indicating the diversity of app users. Øksnebjerg et al.^42^ highlighted that due to personal and contextual features and the large variation in peoples’ needs, the support users require differs considerably. Some studies reported challenges for carers that could impact their ability or willingness to engage with an app. For example, some apps required family members to populate an app to make it individualised and this can be time-consuming.^17,44^ A risk of distress for the carer was also reported by Ryan et al.^17^ as some carers found it difficult to engage with memories that did not include them or were unpleasant to recall. In addition, questions asked whilst using an app highlighted challenges experienced by the person living with dementia which in turn was distressing for the carer.^40,45,46^ This reinforces the need to support both the person living with dementia and the carer not only just at the introduction of an app but also during the continued use of an app.

Another aspect for consideration is how an app integrates into everyday life for the person living with dementia. Some studies highlighted a difficulty integrating the use of apps due to a lack of time^44^ or resources, such as access to a device and to a stable Internet connection.^47^ McAllister et al.^44^ found that this was particularly challenging within a care setting where it is difficult to embed the use of apps into existing care processes due to workload and time constraints and when family members were not available to assist with use. In addition, some individuals did not wish to adopt an app, they expressed a preference for non-technology interventions or off-the-shelf technology,^42,44^ and therefore, the chances of acceptance and subsequent adoption are unlikely.

Rai et al.^47^ suggested the importance of considering diverse user backgrounds when introducing an app. In their study, an app iCST – an app designed for cognitive stimulation therapy (developed in England) was introduced to a group of users (people living with dementia, carers and healthcare professionals) in Indonesia. They discovered that there were limited activities that participants could engage in due to differing cultural contexts and language barriers. The authors concluded that an app needs to be contextualised within Indonesian culture for people to be able to use it. If the users are unable to use an app this will impact adoption negatively.

### Theme 2: Perceived value

From a user's perspective, engagement with an app is influenced by their belief that an app adds value to their day-to-day life, and it is perceived as useful. Many benefits of using apps have been highlighted throughout the studies. The literature review revealed that when participants spoke of the value and benefits of using a particular app, they often commented on how an app made them feel. Reports of positive emotions and feeling happy,^40,47^ feeling safe and secure,^17^ feeling relaxed and having a sense of calm^17,41^ and feelings of enjoyment^40,43^ in addition to feelings of confidence, empowerment, independence and increased self-esteem^41,43,44,45^ were all valued by users. Carers also remarked on how using an app combatted their feelings of loneliness or isolation.^17,41^

The importance of pleasurable experiences which are meaningful to the individual appears as key to successful user engagement. Some studies highlighted that an app was meaningful in their lives and provided mental stimulation^17,47^ or enabled them to try out new things.^41^ Others felt that an app helped them to meet their spiritual needs (to listen to prayer)^47^ or prevent boredom.^47^ This suggests the importance of focusing on aspects that are likely to encourage their interest. Some studies explored this idea further and found that personalised or individual material that is specific to the person living with dementia enhanced their interest and therefore their engagement with an app.^17,43,44,46^

Other studies reported benefits associated with increased communication and interaction between a person living with dementia and their carers, families or friends.^17,40,43–47^ Communication is not only limited to the information or topics on an app but also there are multiple reports of increased communication pertaining to topics outside the use of an app.^41,45^ Ekstrom et al.^45^ explored changes after a communication app was used and found that not only did an app facilitate new conversation but the length of conversations also increased. Many of the studies reported that increased communication led to reflection about positive things in life, a reconnection with past interests or experiences and bringing back ‘happier times’.^41,43,44^ The increased communication resulted in a better understanding of the person living with dementia,^17,41,44^ increased feelings of connection^17,41,46^ and helped to build the relationship between the person living with dementia and their carers.^17,41,44,46^ Similarly, McAllister et al.^44^ and Gall et al.^46^ found that the use of an app in professional settings helped staff to get to know the person living with dementia and facilitated trust between family carers and professional caregivers. The importance of the carers’ experience of using an app is highlighted within the literature. Some studies found that if carers perceived an app as beneficial, they were more likely to encourage a relative with dementia to use it increasing the likelihood of adoption.^17,40,44,45,47^

### Theme 3: Design and content of app

The literature review indicated that both the quality of design and content of an app can impact user adoption. When considering app content, people living with dementia found it is beneficial if an app had rich information on dementia^41,47^ and guidance for caregivers on how to best support people with dementia.^42^ In the study by Bielsten et al.,^41^ participants explained how they liked that they could use an app for ‘tips and advice’ for their current situation. They also valued practical suggestions that may be helpful in the future. They explain how they felt cared for through this support and that ‘someone’ had made an effort to help them in their daily life.

Bielsten et al.^41^ and Ryan et al.^17^ both reported a relationship between users’ positive experiences and a strengths-based app that focused on what remained or could be gained instead of losses or potential problems. Bielsten et al.^41^ discussed the DemPower app, a ‘couple-management app’ that presents video clips of couples sharing their experiences of living with dementia as well as activities such as games, links to valuable information, writing reflections and discussing emotions. Participants commented on how the content of an app encouraged participants to live in the moment, to focus on what the individual can do at that time as opposed to what they may not be able to do in the future. They explained this which was a pleasant change from other resources which focused on ‘identifying and teasing out problems rather than on health-promoting activities’^41 (p.174)^.

Studies suggest that when an app can be personalised to the person living with dementia this can impact their engagement and adoption of an app.^17,43,44,46^ Adding personalised material that is specific to the person living with dementia had many benefits and facilitated a person-centred view,^44^ helped shared special memories^43^ and made it ‘an enjoyable way to care’.^17^ In contrast, Tyack et al.^40^ found when an app was introduced with generic content that was not personalised, which at times the individual did not engage. Some participants highlighted concerns and fears over online safety and security when uploading personal information or memorabilia.^17,42,46,47^ It is important that the person living with dementia and their carer feel safe when using an app.

The design of an application and its ease-of-use is another feature that can impact adoption. In the studies reviewed, some participants report an app was easy to use.^17,43,44^ A participant using the InspireD app^17^ commented on how she liked the straightforward design. ‘It was simple. It was nice and easy . . .’ interestingly this participant also commented on how that ‘if it had’ve been something more complicated I think we would’ve lost interest’ reinforcing the idea that ease of use will impact the adoption of the app. Other studies highlight the importance of providing clear instructions to ensure ease of use.^46,47^ McAllister et al.^44^ expand upon this and comments on the usefulness of paper-based instructions to support the use of the Memory Keeper app.

### Theme 4: Digital literacy and confidence

The ability to operate digital technology was highlighted by numerous studies as having an impact on the adoption of an app.^17,44,46,47^ Throughout the studies, there is evidence of mixed skills and ability to use digital technology, with some individuals having limited issues and others unable to use digital technology at all. Rai et al.^47^ suggest a contributing factor could be a lack of experience with technology prior to diagnosis; however, Øksnebjerg et al.^42^ challenge this idea as the author investigated factors influencing adoption and non-adaptation with people living with dementia and found no significant differences when it came to how much experience they had prior to diagnosis.

Some studies also highlighted a fear of failure or a lack of confidence in the initial stages of using an app.^17,46^ However, over time the person living with dementia reported increased confidence,^17^ competence,^17,40,45^ independence^41^ and a newfound passion for technology.^43^ Ekstrom et al.^45^ reported how this resulted in a reduction in the need for carer support over time. Carers’ lack of digital literacy and confidence in using technology has also been identified as a barrier to using an application.^17,44^ Rai et al.^47^ found to overcome this barrier some participants reached out to either their children or grandchildren as they were considered more ‘technology savvy’ whilst others tried to find a solution themselves or switched off the piece of technology if the problem persisted resulting in non-usage.^47^

## Discussion

Within the selected studies, some authors reported how a person's current health and health-related challenges impacted their engagement with an app. It is important for developers to be aware of the difficulties technology can present, particularly if aspects of the design and content are considered intimidating or challenging. This recommendation has been endorsed in the ‘Best Practice Guidance Human Interaction with Technology in Dementia’ published by the INDUCT and the DISTINCT network^48 (pp.25)^ highlighting that ‘technology should be developed specifically on the characteristics of the person with dementia, with respect to vision, auditory and cognitive capacities’. The need for an inclusive design that addresses usability to reduce the level of challenge is well-documented within the literature.^49^ The degree of usability is extremely important as this will influence the user’s experience, which in turn will impact their level of motivation to continue using an app.^50^ A study by Gibson et al.^49^ (p. 26) explains that ‘Usability is measured in terms of how easily a system can achieve its goals and how efficiently a user can interact with the system through its user interface’. They explain that standard methods to measure usability are observation; concurrent thinking-aloud; single-ease questions; recording by video and/or audio; and the systematic usability scale which is a post-test survey. Boyd et al.^51^ investigated how users perceived the value of the InspireD app (www.theinspiredapp.com) in terms of its usability by using eye tracking. This provided additional information about how the person living with dementia viewed the app, demonstrating how eye tracking can be a useful method for gaining insight into the user experience of a person living with dementia. However, it is important that the methods used are appropriate, suitable and accessible for use by people living with dementia and do not cause distress to the users.^49,50^

Some studies also suggest challenges with usability may be due to a lack of previous experience with technology, however, as there is an increase in the use of everyday technologies and the use of ubiquitous apps increases in popularity, this may not be a barrier in years to come. In fact, there is a possibility that apps targeted for older adults will need to evolve over time, as abilities to use digital technology advance.^52^ The findings presented to highlight that not only can people living with dementia learn to use mobile technology but this can also bring a sense of achievement, self-efficacy and empowerment. Previous assumptions that people living with dementia could not learn how to use touchscreen devices have been invalidated by research, and some studies suggest touchscreens have made computing more accessible for people with dementia as they remove the level of hand-eye coordination needed to operate a mouse and a monitor is not required.^53,54^ A focus on what is possible and the potential for new learning may be a better way forward in terms of enhancing the uptake of apps.

Engaging with people living with dementia as experts regarding their own experiences plays a crucial role in understanding design features that address usability, and therefore, adoption.^55,56^ Despite the importance of adoption, this is often overlooked during the process of design.^24^ Smeenk et al.^57^ suggested that design team members often find it difficult to collaborate with users who have different abilities from them and live in difficult situations and often feel that they lack the necessary skills and experiences to co-design with older users. In addition, challenges such as resources (budget and time) often do not allow all the team members to join co-design sessions which means that some team members cannot work with users and immerse themselves in user situations.^57^ This can make it difficult for designers to be receptive, inclusive and committed to people with dementia. Fennell^58^ explains how app developers should question and reflect upon preconceived ideas regarding dementia to enable them to have empathy and develop technology that is sensitive to the needs of people living with dementia.

Joddrell and Astell^53^ explored how the implementation of tailored settings can impact the accessibility of touchscreen apps for people living with dementia. They found a reduction in usability problems and reported a significant increase in independent initiation when using the app, highlighting how adaptable settings could impact self-reliance and self-efficacy. Adaptable settings and the option to adapt difficulty levels may be beneficial in addressing challenges associated with app adoption in keeping with the progressive nature of the condition, changing needs and the care trajectory.^59^ However, there are some notable challenges with these types of settings including an increased role for a carer to manually tune and adapt the device to the needs of the person living with dementia as well as an increased financial cost to app developers.^60^ The ReAct app^42^ was designed with adaptable features, however, only a limited number of participants made use of the adaptable features. This suggests that further studies are required to explore adaptable features and how they can be implemented in a way that meets the user's needs.

In keeping with previous findings,^61^ people living with dementia are not inclined to adopt technology simply because it is current, the technology must have a perceived value. This literature review suggests that apps that are associated with an emotional reward are more likely to be accepted and adopted. Positive (feeling happy, feeling safe and secure, feeling relaxed and having a sense of calm and enjoyment) and negative emotional responses (lack of self-efficacy, privacy issues or a lack of trust about using new technology) have been reported in this review. Perceived value can be defined as a trade-off between the benefits and costs felt by the user of a product .^62,63^ In this context, for users to perceive an app as valuable, the positive emotions and ‘feel good moments’ must outweigh the negative experiences. Methodologies such as Ecological Momentary Assessment (EMA) can be used to gather accurate data on how an app makes a person living with dementia feel ‘in the moment’ when using an app. This approach provides a high degree of ecological validity as the data are collected in real-world environments.^64^ As EMA requires participants to respond to questions at a given moment, it avoids recall bias which makes it a useful tool for people with memory impairment. Potts et al.^65^ explored user engagement with EMA within the InspireD reminiscence app. The inspireD APP was designed by and for people living with dementia and their carers to promote a more personalised reminiscence experience and showed improvements in the quality of relationships and well-being.^17,66,67^ In the study by Potts et al.^65^ questions on mutuality (the degree of closeness between the person living with dementia and carer)^68^ were presented to users at various points while they were using the InspireD reminiscence app. Overall engagement with EMA was high, suggesting that this method could be a successful way to capture real-time data on how the person living with dementia feels when using an app.

The findings of this review suggest that people living with dementia are more likely to adopt an app that is meaningful to them. The literature suggests the examples of apps that are considered to provide meaningful activity for people with dementia; however, the utility of compiling a list of ‘meaningful activities’ is limited, as the meaning ascribed to specific activities and reasons for participating in them will vary between individuals.^69^ It is, therefore, more useful to understand what makes an activity meaningful for the person. This will encourage a more person-centred approach to identifying ‘meaningful activities’ and supporting the involvement of people with dementia. App libraries such as ‘apps4dementia’ (apps4dementia.orcha.co.uk) or ‘AcTo Dementia’ (www.actodementia.com) can be a valuable resource for people living with dementia to find and identify safe, evidence-based apps which are meaningful to them and can potentially help meet their needs.

Studies included in this review highlighted the importance of introducing an app at the correct time as a key component for adoption. Reviewed studies suggest introducing an app too early or too late may impact acceptance, and therefore, adoption of an app.^42,43^ The Technology Readiness Index, a 36-item scale to measure technology readiness – defined as ‘people's propensity to embrace and use new technologies’^70 (pp.59)^ has previously been used in research involving people living with dementia.^71^ Parasuraman's study recognised that there are both positive and negative feelings that describe the domain of technology readiness^72^ and places focus on the dimensions of optimism, innovativeness, discomfort and insecurity. Whilst this may provide some information on readiness, variables such as mood, behaviours and cognition can fluctuate in people living with dementia which may impact these dimensions. For this reason, further research is required on technology readiness for people living with dementia.

This literature review highlighted the challenges of adopting an app when it did not fit into already established routines. Mulvenna et al.^73^ found the most popular times that people living with dementia and carers preferred to use the InspireD reminiscence app, was around 11 am, 3 pm and 8 pm. These times correspond to post-breakfast, post-lunch and post-evening mealtimes, suggesting embedding an app into already established mealtime routines was beneficial. In contrast, McAllister et al.^44^ found that introducing an app into already established routines was particularly challenging within care settings due to barriers such as time and workload constraints. O’Neill et al.^74^ highlighted that some care home environments can be restrictive and can have regimented practices, as a result maintaining continuity between the older person's past and present role can be challenging. Given this challenge, the use of an app within this setting may be extremely valuable to support both social and emotional needs; however, there is a need to consider readiness to change and challenge current practice within care settings to support app adoption and ongoing engagement.

Other challenges associated with adopting apps into daily routines included access and ability to use mobile applications (e.g., possession of technological devices) as well as access to infrastructures to support their use (e.g., internet connection, battery charging facilities, carer support). This can be particularly challenging in rural areas. Evidence suggests that there is ‘significant if not extreme differences between rural and urban populations in health coverage and access at global, regional, and national levels’.^75^ According to the Global System for Mobile Communications^76^ internet connectivity varies significantly by socio-economic groups and by country income level, with 94% of the ‘unconnected’ population living in low- and middle-income countries. Suggesting that there are barriers to obtaining and retaining access to goods and services such as digital health technologies due to internet access. Within some rural western societies internet access also presents as a barrier. Although there have been some improvements to internet access, rural areas tend to be dependent on low-tech Internet access, with lower speeds and less reliable connections.^77^ The World Health Organisation has a range of global strategies to increase access to rural health care. Mobile technologies have diffused through developing countries quicker than many expected and therefore it is logical that healthcare delivery should build upon these technologies to extend care services into isolated rural areas of developing countries. However, integrating apps into rural healthcare systems remains a challenge due to practical issues such as a lack of material resources, electricity and trained healthcare workers.^78^

The findings presented suggest for an app to be adopted, it should be contextually relevant and include considerations of religion, nationality, cultural beliefs and gender. According to Alzheimer's Society^79^ (n. pag.), ‘It is estimated that 3% of people living with dementia are from black, Asian and minority ethnic (BAME) communities. This equates to around 25,000 people’. This poses a challenge, as people from different cultures may experience different barriers to and facilitators of using the app.^80^ Although only one author in this review explored the concept of cultural relevance when adopting an app,^47^ this finding is in keeping with other studies.^80–83^ These findings shed light on the significance of considering diversity to increase the likelihood of adoption by the users who are likely to have different cultures, languages and ages than the app developers. However, the potential group differences have rarely been explored in app usability and evaluation studies.^81^ It can be argued that integrating a diverse group of people living with dementia (e.g., from different cultural and socio-economic backgrounds, urban and rural environments, different age ranges, experience with digital technology etc.) into the design would increase their acceptance of the technology,^48,81,82^ and therefore, should be prerequisite in designing an application.

The findings demonstrate how some people with dementia required support to operate an app and that the required level of support varied considerably from person to person. People living with dementia may need more explanation and a longer learning time, due to symptoms associated with dementia. Within previous studies, some carers explained how the use of tablets and apps by a relative living with dementia placed additional demands on their caring role,^83^ whilst others believed that it enhanced the quality of their caregiving relationship. This emphasises the need to support carers as well as the person living with dementia at all stages of app adoption, from initial use to continued engagement.

It is important to recognise that some people living with dementia may not wish to engage with digital health apps and may prefer non-technology interventions, off-the-shelf technology or the use of webpages. For example, Øksnebjerg et al.^42^ suggest that instead of using the ReAct app comprising of a calendar that interacts with other features (e.g., diary notes, contacts, checklists and memos) some participants had a preference to use the calendar app that is preinstalled in the device, and others preferred to continue using a paper diary. It is important to avoid excluding people living with dementia who are not interested in adopting applications and service developers should provide alternative non-ICT options when they deliver services and interventions that rely on smartphones, tablets and computers. This involves allowing people to flourish in their own way by acknowledging their individual interests, potential abilities, skills, aspirations and promoting choice. It is important that skills outside the use of apps and touchscreens are not de-valued as often they are related to personal identity, self-esteem and social role. Due to the accessibility of mobile apps that serve a multitude of functions and meet a range of needs, there is a risk of overuse of technology. It is important that the use of touchscreen devices do not replace or overpower other activities that are important for the person living with dementia. Technology may not always offer the right or best possible solution and authors such as IJsselsteijn et al.^55 (pp. 37)^ explain that ‘the world that surrounds us and shapes our perceptual-motor experiences, however, is not confined to a smooth, cold, light-emitting, glass pane’, suggesting that if the same or better results can be achieved by non-technological means, this should be explored as it may be of greater benefit to the person living with dementia.

## Conclusion

People are diverse and so are their reasons for the adoption of apps. This review provides insight into the range of factors that impact the adoption of apps for people living with dementia. We present four themes: Theme 1: Personal and contextual factors; Theme 2: Perceived value; Theme 3: Design and content of app; and Theme 4: Digital Literacy and Confidence. Understanding the factors that impact the adoption of mobile applications is critical to their success and these findings can be beneficial for app developers and for people living with dementia and their carers. The findings discussed should be considered as a prerequisite in designing an application for people living with dementia and throughout the Technology Acceptance Lifecycle continuum^24^ to reduce the risk of non-usage.

### Strengths and limitations

This paper reports a robust approach to a literature review on the factors impacting the adoption of mobile applications (apps) for people living with dementia. It provides readers with a replicable approach in the search of the literature, with specified search terms, and inclusion and exclusion criteria used to limit bias in the selection of articles. The use of the MMAT also provided a way of assessing the suitability and the quality of papers included in the review. Each paper was read by two reviewers to ensure the accuracy of information recorded and assessed. Papers included in this review are limited to mainly Global North countries (Table 3), findings may not be applicable to the needs and experiences of users from other regions. Some studies included in the review had a small number of participants (e.g., Ekstrom et al.^45^ *n* = 2) which can impact generalisability; however, rich in-depth data were collected. Although every effort was made to identify all relevant papers in this review, the decision to exclude secondary sources may have been limiting and future reviews should consider their inclusion, especially considering the growing interest in the use of mobile applications. As there is a dearth in the literature pertaining to factors impacting the adoption of apps in people living with dementia, this review includes findings from published empirical research on the use of apps. The researchers identified underlying ideas and conceptualisations to inform the semantic content of the review this may introduce an element of bias.

### Implications for practice, policy and research

The acquired information can be used by people living with dementia, carers, healthcare providers, policy makers and app developers to maximise the likelihood of adoption. Besides these theoretical contributions to the field digital health literature, these findings may also present vital information for the development of policies and guidelines that will become helpful for the implementation of digital health apps successfully. As the availability digital health applications is growing and there are increased opportunities for the embedding of digital health apps within existing health systems.^3,4,6^ To do so, there is a need for a digital first policy^84,85^ for people living with dementia, that is user-focused, inclusive and sustainable. The findings within this review can inform this policy and add to what is already known to ensure the design, development, testing and implementation of an app is based on the users’ needs to enhance the likelihood of adoption.

Timely identification of the factors discussed within this review may prompt intervention and increase the likelihood of adoption. The findings presented may also aid health care professionals when considering the appropriateness of an app and the likelihood of a person living with dementia adopting an app. Our study's findings could enhance the likelihood of successful technology adoption and implementation by considering how a person living with dementia can be supported when using apps. This learning is critical for app developers as an understanding of the potential range of factors that impact the adoption of apps for people living with dementia is important for innovation-ready design.

As the research base on technology acceptance in people living with dementia is minimal, there are opportunities for future research in this area. Additional qualitative research involving people living with dementia to capture their perspective, learn about their diverse lived experiences with mobile apps and consider their viewpoint would be beneficial. The importance of engaging with people living with dementia as experts regarding their own experiences plays a crucial role in understanding design features that address usability, and therefore, adoption. The majority of studies included in this review are snapshots of app use, as app acceptance is an ongoing process, longitudinal research is needed to evaluate factors that impact adoption over time. Future research should consider the factors in other geographies/cultures, time frames and contexts including socio-economic factors. It would also be very relevant to include some non-users to the participants' mix to cover their views as well.
